# The Role of Markers of Inflammation in Traumatic Brain Injury

**DOI:** 10.3389/fneur.2013.00018

**Published:** 2013-03-04

**Authors:** Thomas Woodcock, Maria Cristina Morganti-Kossmann

**Affiliations:** ^1^Australian School of Advanced Medicine, Macquarie UniversitySydney, NSW, Australia; ^2^Department of Epidemiology and Preventive Medicine, Australian and New Zealand Intensive Care Research Centre, Monash UniversityMelbourne, VIC, Australia

**Keywords:** traumatic brain injury, biomarkers, inflammation, cytokines, chemokines

## Abstract

Within minutes of a traumatic impact, a robust inflammatory response is elicited in the injured brain. The complexity of this post-traumatic squeal involves a cellular component, comprising the activation of resident glial cells, microglia, and astrocytes, and the infiltration of blood leukocytes. The second component regards the secretion immune mediators, which can be divided into the following sub-groups: the archetypal pro-inflammatory cytokines (Interleukin-1, Tumor Necrosis Factor, Interleukin-6), the anti-inflammatory cytokines (IL-4, Interleukin-10, and TGF-beta), and the chemotactic cytokines or chemokines, which specifically drive the accumulation of parenchymal and peripheral immune cells in the injured brain region. Such mechanisms have been demonstrated in animal models, mostly in rodents, as well as in human brain. Whilst the humoral immune response is particularly pronounced in the acute phase following Traumatic brain injury (TBI), the activation of glial cells seems to be a rather prolonged effect lasting for several months. The complex interaction of cytokines and cell types installs a network of events, which subsequently intersect with adjacent pathological cascades including oxidative stress, excitotoxicity, or reparative events including angiogenesis, scarring, and neurogenesis. It is well accepted that neuroinflammation is responsible of beneficial and detrimental effects, contributing to secondary brain damage but also facilitating neurorepair. Although such mediators are clear markers of immune activation, to what extent cytokines can be defined as diagnostic factors reflecting brain injury or as predictors of long term outcome needs to be further substantiated. In clinical studies some groups reported a proportional cytokine production in either the cerebrospinal fluid or intraparenchymal tissue with initial brain damage, mortality, or poor outcome scores. However, the validity of cytokines as biomarkers is not broadly accepted. This review article will discuss the evidence from both clinical and laboratory studies exploring the validity of immune markers as a correlate to classification and outcome following TBI.

## Introduction

Traumatic brain injury (TBI) has long been called a “silent epidemic” (Goldstein, 1990; Coburn, 1992). In the United States, it accounted for at least 1.5 million emergency room visits and hospitalizations annually between 1995 and 2001, a number that has certainly increased as a result of the wars in Iraq and Afghanistan (Langlois et al., 2006). Many survivors of TBI are left with long term disabilities, and even a mild TBI can leave people with cognitive impairments, difficulty in concentrating, fatigue, and headaches. It disproportionately affects young men, but is also increasingly common in the elderly population (Hukkelhoven et al., 2003). The financial burden to the United States has been estimated to exceed $56 billion annually (Finkelstein et al., 2006; Langlois et al., 2006; Rutland-Brown et al., 2006) while in 2008 alone it has been calculated to approximately AU$ 8.6 billion in Australia. Despite advances in prevention measures, surgical, and diagnostic techniques, there have been relatively few changes in the way TBI patients are managed, and so far no pharmacological treatment has been found to confer neuroprotection by targeting secondary injury mechanisms.

Currently, the main focus for TBI patient management is monitoring and maintenance of normal intracranial pressure (ICP), and cerebral perfusion pressure (CPP). One of the effects of elevated ICP is the reduction in CPP, which consequently leads to secondary ischemia. It is no surprise therefore that high ICP has been shown to be associated with mortality and poor outcome in TBI patients (Narayan et al., 1982; Treggiari et al., 2007). Although treatment of high ICP is certainly beneficial, being able to predict elevations in ICP and apply preventive or early interventions would be more ideal. At present, there is no method with which to predict changes in ICP, since neurological examinations and routinely administered computed tomography (CT) scans do not provide this information. To this end, the development of biomarkers that have predictive accuracy with regard to ICP would greatly improve patient management.

In addition to lowering elevated ICP, treatment and management of a TBI patient could depend on a wide range of factors. Human TBI is a very heterogeneous condition due to the intrinsic combination of focal and diffuse injuries and the individual response via secondary mechanisms of neurodegeneration. Understanding these processes would aid the development of an individual patient’s tailored treatment plan. At present patients are categorized based on admission characteristics including age, pupil reaction, Glasgow coma scale scores (GCS), body temperature, blood glucose, non-cranial injuries (Hukkelhoven et al., 2005; Mushkudiani et al., 2008), and also observations from a CT scan (e.g., Marshall CT classification, primarily prognostically oriented Rotterdam score; Marshall et al., 1992; Maas et al., 2005). Magnetic resonance imaging (MRI) is routinely used in the clinic and is becoming very popular for the quantification of brain damage particularly in diffuse TBI. The more recently developed diffusion tension (DT)-MRI can detect with in great detail alterations in the microstructure of the white matter and shows considerable promise in the assessment of axonal damage (Salmond et al., 2006). However, these neuroimaging techniques reveal little or no information regarding secondary injury processes such as excitotoxicity, neuroinflammation, blood-brain barrier (BBB) breakdown, ischemic damage, and cell death. Biomarkers promise to arm clinicians with all this additional, patient-specific information. In the context of this review a biomarker is a molecule that can be measured in a patient, which reflects the pathology of TBI, how the pathology is likely to develop, and become predictive factors for long term neurological outcome. It would be of great benefit to the patient if an endogenous molecule could be identified to have an expression profile, which can be linked to the type or extent of secondary injury. With this information clinicians would be able to make more informed decisions regarding treatments most likely to lead to an optimal outcome. Furthermore, detection of early biomarker levels would be invaluable in identifying patients that are most likely to benefit from a specific experimental treatment. Indeed, the diverse nature of human TBI has been cited as a potential explanation for lack of a successful clinical trial (Statler et al., 2001). While it is now clear that there is no “silver bullet” with which all TBI patients can be treated, categorization of TBI patients using biomarkers in combination with traditional methods might allow for specific treatments to be given to those most likely to benefit.

Identifying secondary injury mechanisms may also be useful in determining the type of injury that a person has received and the degree of its progression in the long term. For example, the “War on Terror” has led to an increase in the incidence and awareness of blast injuries, which involve a rapid change in air pressure around the body. Blast injury is becoming increasingly common, yet symptoms often do not manifest until weeks or months after the incident (Zeitzer and Brooks, 2008). Even a mild TBI can leave survivors with long term cognitive deficits and behavioral problems, which impact on their daily lives (Corrigan et al., 2004; Pagulayan et al., 2006; Strandberg, 2009). Being able to specifically predict which patients are likely to develop these neurological symptoms would enable doctors to make referrals to specific rehabilitation programs, council patients and family members, and encourage vigilance in reporting such changes.

## The Development of Biomarkers in TBI

The most commonly used biomarkers include S100B, neuron-specific enolase (NSE), and myelin basic protein (MBP; Palfreyman et al., 1978; Thomas et al., 1978) reflecting the extent of tissue damage as well as having prognostic value for long term outcome (Baker et al., 2009; Svetlov et al., 2009; Kovesdi et al., 2010). There are several observational clinical TBI studies where S100B has been successfully correlated with initial brain injury severity (GCS), size of brain damage (on CT/MRI scans), and neurological outcome (Glasgow Outcome Scale/Extended; GOSE). However, it is only recently that biomarkers are being employed in the context of clinical trials with prospective collection of physiological data, outcomes, and the clinical assessment of efficacy of an intervention. The ultimate aim is to demonstrate the correlations between a drug’s neuroprotection and the reduced concentration of biomarkers in TBI patients. Biomarkers are defined as sensitive early measure of outcome than the currently available neurological scores assessed only 6 months after TBI. In addition due to the common problem of TBI clinical trials being inadequate in patient numbers, the use of biomarkers could provide a more powerful tool to detect outcome differences in TBI patient populations.

At present, the most studied TBI biomarker is S100B, a low-molecular weight calcium binding protein secreted by astrocytes. The validity of S100B as a potential TBI biomarker relies on its constitutively low expression in serum and cerebrospinal fluid (CSF), which is rapidly released into the CSF and serum following brain injury. A very strong correlation of S100B levels and severity of injury has been reported (Savola et al., 2004), as well as a link between high S100B expression and unfavorable outcome (Herrmann et al., 2001; Townend et al., 2002; Vos et al., 2004; Rainey et al., 2009). However, S100B is not ideal as a TBI biomarker because it does not readily cross the BBB, its serum levels increase after peripheral trauma in the absence of brain injury (Anderson et al., 2001; Savola et al., 2004; Torabian and Kashani-Sabet, 2005), and it has not always been found to reliably predict outcome (Berger et al., 2007; Piazza et al., 2007). In the search for a reliable biomarker of TBI, other molecules have been assessed, including glial fibrillary acidic protein (GFAP), NSE, MBP, α-II-spectrin breakdown products (BDPs), ubiquitin C-terminal hydrolase-L1, and various cytokines (Dash et al., 2010; Schiff et al., 2012).

## The Potential of Inflammatory Cytokines as Biomarkers of TBI

One of the integral features of TBI is the inflammatory reaction initiated and regulated by an array of pro- and anti-inflammatory cytokines. Cytokines are small, short-lived proteins produced by blood leukocytes and glial cells. They are quickly released in response to TBI and are rapidly sequestered. There are a large number of different cytokines, many with overlapping functions that form a complex network of inflammatory mediators. Cytokines that initiate or propagate an inflammatory response are said to be pro-inflammatory, while cytokines that inhibit the inflammatory response are called anti-inflammatory. The expression profile of each cytokine following brain injury has the potential to provide information about the extent of tissue damage, and can be easily and rapidly measured via immunological assays. However, cytokine concentrations vary depending on the tissue or fluid they are measured in (brain tissue, CSF blood, serum, plasma, etc.), and the temporal profile of the cerebral immune response in rodent versus human data can present differences as well as commonality. Some of the most studied cytokines with respect to brain injury and their potential as biomarkers are discussed in this review. A summary of relevant literature is shown in Table 1.

**Table 1:** **Studies relevant to the development of cytokines as biomarkers of TBI**.

Cytokine	Species	Injury/model	Tissue/fluid	Findings	Reference
IL-1β	Rat	LFP, weight-drop	Brain homogenates	Increase in mRNA expression occurs within 1 h and peak mRNA and protein expression is between 12 and 24 h after injury	Fan et al. (1995), Kinoshita et al. (2002), Lu et al. (2005a), Lu et al. (2005b), Kamm et al. (2006)

	Rat	Weight-drop	Plasma	No change in IL-1β expression following TBI	Kamm et al. (2006)

	Rat	LFP	Brain homogenates	mRNA expression of IL-1β is higher in severe versus moderate injury severity	Kinoshita et al. (2002)

	Rat	DAI-hypoxia	Brain homogenates	Increased and prolonged IL-1β expression when TBI is combined with post-traumatic hypoxia	Yan et al. (2011)

	Human	TBI	Post-mortem tissue	Increased mRNA expression within minutes of injury	Frugier et al. (2010)

	Human	TBI	CSF and serum	Peak expression of IL-1β in CSF and serum following TBI is very low	Kossmann et al. (1996), Kossmann et al. (1997), Singhal et al. (2002), Hergenroeder et al. (2010)

	Human	TBI	Serum	IL-1β levels within 6 h of injury correlate with GCS	Tasci et al. (2003)

	Human	Severe TBI and pediatric TBI	CSF	Elevated IL-1β expression associated with poor outcome and increased ICP	Hayakata et al. (2004), Chiaretti et al. (2005), Shiozaki et al. (2005)

	Human	TBI	Brain parenchyma, CSF, serum	No correlation of IL-1β expression with ICP or outcome	Winter et al. (2004), Stein et al. (2011)

IL-1β/IL-1ra	Human	Severe TBI	Brain parenchyma	High IL-1ra/IL-1β ratio is associated with better outcome	(Hutchinson et al. (2007)

TNF	Rat	TBI	Brain homogenates, brain slices	Increased mRNA and protein expression detectable at 1 h, and peak expression between 4 and 8 h post-TBI	Taupin et al. (1993), Shohami et al. (1994), Fan et al. (1996), Knoblach et al. (1999), Dalgard et al. (2012)

	Rat	LFP	Brain homogenates	TNF expression increases after severe TBI, but not mild TBI	Knoblach et al. (1999)

	Rat	DAI-hypoxia	Brain homogenates	DAI and post-traumatic hypoxia leads to increased expression of TNF versus DAI alone	Yan et al. (2011)
	Rat	CCI	CSF	Peak levels of TNF in CSF are not reached until 24 h after TBI	Stover et al. (2000)

	Human	TBI	CSF, serum, plasma	TNF is increased in CSF, serum, and plasma following TBI	Goodman et al. (1990), Ross et al. (1994), Morganti-Kossmann et al. (1997), Csuka et al. (1999)

	Human	TBI	Post-mortem tissue	TNF mRNA and protein can be detected in the brain within minutes of injury	Frugier et al. (2010)

	Human	Severe TBI	CSF	TNF protein concentrations peak in the CSF within 24 h	Hayakata et al. (2004)

	Human	Severe TBI	CSF, serum	Six hours after TBI, TNF expression is higher in CSF than in serum. TNF expression does not correlate with outcome	Shiozaki et al. (2005)

	Human	Severe TBI	CSF, serum	Increased serum TNF levels correlate with increased ICP and decreased CPP, but not outcome. TNF concentrations in CSF not linked to ICP, CPP, or outcome	Stein et al. (2011)

IL-10	Rat	LFP	Brain homogenates	IL-10 expression increases rapidly, and remains elevated from 4 to at least 20 h after TBI	Knoblach and Faden (1998)

	Rat	CCI	Brain homogenates	IL-10 expression is reduced in the brains of TBI versus sham animals 1 day after surgery	Lee et al. (2012)

	Human	Severe TBI	CSF, serum, plasma	IL-10 expression in both CSF and serum increases rapidly following TBI, and is higher in CSF than in serum or plasma. There is no correlation of IL-10 with BBB integrity	Csuka et al. (1999), Maier et al. (2001), Woiciechowsky et al. (2002)

	Human	Severe TBI	CSF, serum	IL-10 expression is higher in serum than in CSF following TBI	Hayakata et al. (2004)

	Human	Pediatric TBI	CSF	Increased IL-10 levels in CSF are linked to mortality	Bell et al. (1997)

	Human	Severe TBI	CSF, serum	Increased IL-10 is linked to BBB dysfunction and mortality	Kirchhoff et al. (2008)

	Human	Severe TBI	CSF	IL-10 expression is higher in patients that had an unfavorable outcome	Shiozaki et al. (2005)

	Human	Severe TBI	CSF, serum	No link between IL-10 and outcome	Maier et al. (2001), Woiciechowsky et al. (2002), Lo et al. (2009, 2010), Stein et al. (2011)

IL-6	Rat, mouse	LFP, PBBI, weight-drop	Brain homogenates, brain parenchyma	IL-6 expression is undetectable in normal brain, but increases rapidly, peaking at 2–8 h following TBI	Woodroofe et al. (1991), Taupin et al. (1993), Shohami et al. (1994), Maegele et al. (2007), Williams et al. (2007), Ziebell et al. (2011), Weckbach et al. (2012)

	Rat	CCI, DAI	CSF, serum	IL-6 expression is higher in CSF than in serum. IL-6 expression increases from 1 h and peaks at 2–5 h after injury	Woodroofe et al. (1991), Hans et al. (1999), Stover et al. (2000)

	Rat	CHI, poly-trauma	Serum	IL-6 expression in serum cannot discriminate between peripheral and CNS injuries	Maegele et al. (2007), Weckbach et al. (2012)

	Human	TBI	CSF, serum	Following TBI IL-6 expression increases to a greater extent in CSF than serum	Kossmann et al. (1996), Winter et al. (2004), Hillman et al. (2007), Chiaretti et al. (2008)

	Human	TBI	Plasma	IL-6 concentrations greater than 100 pg/mL are associated with severe TBI	Woiciechowsky et al. (2002)

	Human	TBI	Plasma	Increased IL-6 concentrations correlate with poor outcomes	Arand et al. (2001), Woiciechowsky et al. (2002)

	Human	Pediatric TBI	Serum	No correlation between IL-6 levels and outcome	Kalabalikis et al. (1999)

	Human	TBI	Serum	IL-6 levels within 17 h of injury can be used to predict elevated ICP	Hergenroeder et al. (2010)

	Human	TBI	Brain parenchyma	Higher parenchymal levels of IL-6 correlate with better outcomes	Winter et al. (2004)

	Human	TBI	Brain parenchyma	No relationship between IL-6 and ICP, brain oxygenation, or edema	Perez-Barcena et al. (2011)

IL-8	Rat, mouse	CCI, weight-drop	Brain homogenates	IL-8 functional homologs CXCL-1 and CXCL-2 exhibit peak expression at 4–12 h after TBI	Otto et al. (2002), Dalgard et al. (2012)

	Human	TBI	CSF, serum	Following TBI, increased IL-8 expression can be measured in CSF and to a lesser extent in serum	Kossmann et al. (1997), Morganti-Kossman et al. (1997), Whalen et al. (2000), Kushi et al. (2003a)

	Human	Pediatric TBI	CSF	IL-8 levels following TBI correlate with mortality	Whalen et al. (2000)

	Human	Severe TBI	CSF, plasma	Lower IL-8 levels in plasma are associated with survival. CSF IL-8 levels do not vary between survivors and non-survivors	Gopcevic et al. (2007)

	Human	Severe TBI, pediatric TBI	Serum	Increases in IL-8 after TBI correlate with unfavorable outcome and are associated with mortality	Mussack et al. (2002), Kushi et al. (2003b); Lo et al. (2010)

## Interleukin-1

The Interleukin-1 (IL-1) family of cytokines are key mediators of the inflammatory response peripherally and centrally. The IL-1 related molecules are perhaps the best known in relation to acute TBI, having been widely studied in models of both focal and diffuse injury (Fan et al., 1995; Benveniste, 1998; Yatsiv et al., 2002; Brough et al., 2011). The IL-1 receptor type I (IL-1R) is thought to mediate many of the effects of the IL-1 cytokines, and is expressed on multiple cell types in the brain (Holmin et al., 1997; Csuka et al., 2000; Pinteaux et al., 2002; Lu et al., 2005a). However, there is also evidence to suggest that some of the effects of IL-1 cytokines are independent of the IL-1R (Touzani et al., 2002; Boutin et al., 2003; Loscher et al., 2003). Intranuclear actions of IL-1 to regulate gene transcription and RNA splicing may account for some of these actions (Luheshi et al., 2009).

The IL-1 family includes the closely related agonists IL-1α and IL-1β, the antagonist IL-1ra, and the other family member, the agonist IL-18 (Dinarello, 1994, 1998, 2009; Barksby et al., 2007). Of these, the IL-1β isoform is by far the most often reported in TBI. IL-1β is a pro-inflammatory cytokine and has been implicated in the release of phospholipase-2 (PLA2), prostaglandins, and the activation of cyclooxygenase-2 (COX-2; Chung and Benveniste, 1990; Aloisi et al., 1992; Molina-Holgado et al., 2000; Rothwell, 2003). Furthermore, the primary mechanism of action for IL-1β is believed to be the regulation of release of other cytokines. IL-1β has also been shown to play a role in apoptosis (Holmin and Mathiesen, 2000), adhesion of leukocytes to endothelial cells (Bevilacqua et al., 1985), BBB disruption (Quagliarello et al., 1991), and edema formation (Holmin and Mathiesen, 2000). The fundamental pro-inflammatory and neurotoxic function of IL-1β is demonstrated by studies that have aimed to attenuate IL-1 effects. For example, the antagonist IL-1ra has been found to reduce neuronal damage in rodent brain injury models (Relton and Rothwell, 1992; Yang et al., 1998). Improved cellular and behavioral outcomes from brain injury have been reported in rats treated with recombinant human IL-1ra (Toulmond and Rothwell, 1995), mice lacking the IL-1R (Basu et al., 2002), mice over-expressing IL-1ra (Tehranian et al., 2002), or by means of intraventricular administration of IL-1β or IL-1α antibodies to rats (Lu et al., 2005a,b). The intensive research into IL-1 with regard to TBI has led to it being considered as a biomarker of early neuroinflammation and consequent tissue damage.

### Laboratory evidence

Studies in animal models of focal and diffuse TBI have consistently shown that basal levels of IL-1β are very low (O’Connor and Coogan, 1999; Krueger, 2008), and that an increase in IL-1β expression is detectable as early as 1 h after trauma (Fan et al., 1995; Kinoshita et al., 2002; Lu et al., 2005a,b; Kamm et al., 2006). In rodent brain homogenates peak mRNA and protein expression occurs between 12 and 24 h after injury (Fan et al., 1995; Ciallella et al., 2002; Ahn et al., 2004; Lu et al., 2005a,b, 2007; Kamm et al., 2006; Maegele et al., 2007; Semple et al., 2010a,b; Shojo et al., 2010); and levels of IL-1β mRNA within 24 h of injury do appear to be associated with injury severity (Kinoshita et al., 2002). Despite the lack of direct correlations with brain damage or outcome, we have shown that while IL-1β peaks at 2 h in the rat cortex following a diffuse axonal injury, when combined with post-traumatic hypoxia the expression of IL-1β is significantly enhanced and prolonged (Foda and Marmarou, 1994; Yan et al., 2011). The exacerbated production of IL-1β is therefore important to consider, especially when secondary injuries occur.

### Clinical evidence

IL-1β is barely detectable in the serum or CSF of healthy individuals, and has proved difficult to measure following human TBI (Kossmann et al., 1996, 1997; Hergenroeder et al., 2010). One recent study reported peak IL-1β in CSF of 1.4–25 pg/mL, and serum concentrations of 0.8–7.6 pg/mL (Singhal et al., 2002). More recently, measurements of IL-1β concentrations in post-mortem tissue from TBI patients have confirmed that a global upregulation occurs within a few minutes to hours of injury (Frugier et al., 2010). Similar small increases in IL-1β concentrations have been reported previously in stroke patients (Tarkowski et al., 1995). Although changes in IL-1β expression in CSF and serum following injury appear to be small, attempts have been made to correlate IL-1β levels with outcome. Serum levels of IL-1β taken within 6 h of TBI have been found correlate with GCS in a cohort of 48 patients (Tasci et al., 2003). In other studies in severe brain injury patients high CSF concentrations of IL-1β were associated with poor outcome and increased ICP (Hayakata et al., 2004; Shiozaki et al., 2005). In pediatric TBI, the CSF levels of IL-1β have been correlated with outcome assessed by the Glasgow outcome score (GOS; Chiaretti et al., 2005). Finally, in one study IL-1β and IL-1ra were measured in brain microdialyzates of 15 TBI patients, and better outcomes were reported in patients with a high IL-1ra/IL-1β ratio (Hutchinson et al., 2007). Despite these results, other groups have failed to correlate IL-1β to ICP or outcome (Winter et al., 2004; Stein et al., 2011).

## Tumor Necrosis Factor

Tumor Necrosis Factor (TNF; formerly TNFα) is a multifunctional cytokine most often referred to as a potent pro-inflammatory cytokine, produced by microglia and astrocytes. Early studies mostly in rat models of TBI, administration or inhibition of TNF suggested that increased expression of TNF is detrimental (Ramilo et al., 1990; Kim et al., 1992; Shohami et al., 1996; Knoblach et al., 1999; Trembovler et al., 1999). However, more recent work employing TNF and TNF receptor knockout mice have shown that mortality rates are increased and long term recovery impaired in these models of focal TBI (Scherbel et al., 1999; Sullivan et al., 1999; Stahel et al., 2000). These apparently conflicting data mirror findings from other inflammatory mediators and demonstrate the dual role of TNF as both a pro- and anti-inflammatory cytokine (Shohami et al., 1999; Lenzlinger et al., 2001; Morganti-Kossmann et al., 2002; Schmidt et al., 2005).

### Laboratory evidence

In injured rat brain, increased TNF mRNA can be detected prior to the cytokine protein itself, and upregulation of TNF was shown to precede leukocyte infiltration to the site of injury (Riva-Depaty et al., 1994; Shohami et al., 1997). This suggests that TNF is produced early by resident brain cells in response to neuronal injury. Increases in TNF protein have been measured at 1 h, and peak levels were found between 4 and 8 h after injury (Taupin et al., 1993; Shohami et al., 1994; Fan et al., 1996; Knoblach et al., 1999; Dalgard et al., 2012). Early increases in TNF expression could prove to be useful in the clinical setting as a diagnostic/prognostic factor. In fact TNF was shown to reflect injury severity, since one study using the lateral fluid percussion (LFP) injury reported that while increases could be measured for severe injury, no change in TNF was recorded for a mild injury (Knoblach et al., 1999). Furthermore, in a recent study from our laboratory, we found that TNF is only increased in rats subjected to a combined diffuse brain injury and hypoxia, but not in diffuse brain injury alone (Yan et al., 2011). In contrast to previous published work, when using the closed brain injury model of focal TBI, we did not find increased expression of TNF in brain homogenates over 24 h (Bye et al., 2007; Semple et al., 2010a). Another interesting finding from animal studies is a difference in the timing of TNF peaks in the parenchyma versus CSF of rats. In brains of rats subjected to LFP injury TNF expression peaks at 4–6 h and is resolved by 24 h (Fan et al., 1996; Knoblach et al., 1999), yet in the controlled cortical impact (CCI) model TNF expression in the CSF did not peak until 24 h post-injury (Stover et al., 2000). Whether this represents a differential response to injury type, or a delay in movement of cytokine from parenchyma to CSF remains to be determined.

### Clinical evidence

Following TBI increases in TNF levels have been reported in the CSF and serum of patients (Goodman et al., 1990; Ross et al., 1994). In work from our group TNF concentrations were measured in the CSF and serum of TBI patients at 24 h intervals, and TNF was significantly elevated from controls (Morganti-Kossmann et al., 1997; Csuka et al., 1999). Given that TNF expression in animal models usually peaks and resolves within the first 24 h this is perhaps not surprising. The delayed and sustained increase in TNF measurements in human CSF (3 days to 3 weeks) as compared to rapid fluctuations observed in brain tissue may reflect different mechanisms of cytokine metabolism and degradation in these environments. Indeed, more recently we have shown that TNF mRNA and protein can be detected in post-mortem brain tissue from TBI patients that died within 17 min of injury (Frugier et al., 2010). In another study, Hayakata et al. (2004) examined CSF from 23 severe TBI patients and a peak in TNF of 20–30 pg/mL was recorded within 24 h. They then attempted to analyze the associations of TNF levels with raised ICP and poor outcome (GOS < 4 at 6 months), but no correlation was found. In a subsequent study the same group measured TNF in both the CSF and serum of 35 TBI patients with or without additional injury at exactly 6 h after injury (Shiozaki et al., 2005). They reported that TNF levels were higher in CSF (median 18 pg/mL) versus serum (median 5 pg/mL) regardless of the presence of additional injury. Again, there was no correlation between TNF and GCS, ICP, or neurological outcome. More recently, Stein et al. (2011) analyzed CSF and serum samples from 24 patients at 12 h intervals for 7 days after having sustained a severe TBI. In the same patients ICP and CPP were continually monitored so that associations between cytokine levels and subsequent changes in ICP or CPP could be investigated. They reported that increased serum, and not CSF, concentrations of TNF moderately correlate with subsequent increases in ICP or decreases in CPP. However, they did not find any relationship of any cytokine concentration [IL-1β, Interleukin-6 (IL-6), Interleukin-8 (IL-8), Interleukin-10 (IL-10), or TNF] with outcome (GOS < 5).

## Interleukin-10

Interleukin-10 is regarded to be primarily an anti-inflammatory cytokine, having a potent inhibitory effect on production of several pro-inflammatory mediators including IL-1β and TNF, but also IL-1α, granulocyte-macrophage colony stimulating factor (GM-CSF), IL-6, IL-8, IL-12, and IL-18 (de Waal Malefyt et al., 1991, 1993; Fiorentino et al., 1991; D’Andrea et al., 1993; Gruber et al., 1994). The inhibition of IL-1β and TNF is its most important function, since these cytokines are known to play central roles in initiation and propagation of the inflammatory response. Indeed, rats subjected to LFP injury and treated with IL-10 have improved outcomes and reduced levels of IL-1β and TNF in brain tissues (Knoblach and Faden, 1998).

### Laboratory evidence

Although the anti-inflammatory properties of IL-10 following TBI are well established, there is relatively little information regarding the expression profile of IL-10 following TBI in animals. Using the FPI model, one study found that in the brain IL-10 increased rapidly during the first 4 h following injury and remained elevated for at least 20 h thereafter (Knoblach and Faden, 1998). However, a more recent study reported a reduction in IL-10 expression in brains of rats 1 day after CCI (Lee et al., 2012). No changes instead were shown in diffuse brain injured rats over 4 days post-injury, whether with or without the addition of hypoxia (Yan et al., 2011). This suggests that such anti-inflammatory cytokine may play a role in a delayed phase after TBI. The discrepancy between the two studies indicates that there may be a differential expression profile of IL-10 based on the type of injury, since the weight-drop model produces a diffuse axonal injury in the absence of focal damage, whereas CCI is primarily a focal injury.

### Clinical evidence

We have shown that IL-10 is elevated in the CSF and serum of patients with isolated, severe TBI (Csuka et al., 1999). In the CSF IL-10 was elevated in 26 out of 28 patients (range: 1.3–41.7 pg/mL) versus controls, but in serum only 7 patients displayed elevated IL-10 (range: 5.4–23 pg/mL). The temporal profile was similar in both fluids, exhibiting a rapid early rise and peak followed by a slow decline. In addition to cytokine measurements, BBB function has been assessed in TBI patients using the CSF/serum albumin ratio, and no correlation between the two variables has been found (Csuka et al., 1999; Maier et al., 2001). The lack of association of IL-10 levels with BBB dysfunction, combined with the fact that IL-10 CSF levels exceeded serum levels in most patients suggest an intrathecal origin for this cytokine. However, not all studies have corroborated this hypothesis, since serum levels have been reported to be significantly higher than CSF levels in some studies (Hayakata et al., 2004). The presence of additional injuries could easily account for this difference (Hensler et al., 2000; Dziurdzik et al., 2004; Shiozaki et al., 2005). Other studies have confirmed that in severe TBI IL-10 expression increases early, reaching a peak within 2–8 h of injury (Woiciechowsky et al., 2002). Higher levels of IL-10 have been linked to better outcome in some studies, but not in others. For instance, an early study reported a link between increased IL-10 levels in CSF and mortality in pediatric TBI (Bell et al., 1997), and more recent work in adult severe TBI made a similar link between increased IL-10 and mortality (Kirchhoff et al., 2008). The concentration of IL-10 in the CSF has also been shown to be higher in patients that have an unfavorable outcome (GOS < 4) assessed 6 months after injury (Shiozaki et al., 2005). However, other studies have failed to find any connection between IL-10 and outcome (Maier et al., 2001; Woiciechowsky et al., 2002). In pediatric TBI, Lo et al. measured serum IL-10 levels on day 1, and found they could not differentiate severe and non-severe injury or predict favorable outcome (Lo et al., 2009), even when paired with GCS (Lo et al., 2010). In a more recent study there was no correlation of IL-10 in serum or CSF with outcome assessed at 6 months using the GOSE scores (Stein et al., 2011). The IL-10 response to peripheral injuries as reported in multi-trauma patients could be part to blame for the difficulty in making associations between TBI variables and IL-10 levels (Shimonkevitz et al., 1999; Hensler et al., 2000; Dziurdzik et al., 2004; Shiozaki et al., 2005).

## Interleukin-6

Interleukin-6 has been extensively studied, and has been found to be involved in a large number of physiological and pathophysiological processes. IL-6 is known to regulate inflammation, immunity, bone metabolism, hematopoiesis, and neural development (Romano et al., 1997). In addition, a role for IL-6 has been implicated in aging, osteoporosis, autoimmune disease, Alzheimer’s disease, and brain injury. Although IL-6 is not exclusively expressed in the CNS, it does exhibit a significant upregulation following brain injury (Morganti-Kossmann et al., 1992; Kossmann et al., 1995).

### Laboratory studies

Laboratory studies have shown that in the brain IL-6 is expressed by astrocytes (Benveniste et al., 1990; Van Wagoner and Benveniste, 1999), microglia (Woodroofe et al., 1991; Sebire et al., 1993), and neurons (Schobitz et al., 1993; Gadient and Otten, 1994; Ringheim et al., 1995; Sallmann et al., 2000). It inhibits the synthesis of TNF (Aderka et al., 1989), induces synthesis of nerve growth factor (NGF; Kossmann et al., 1996), inhibits *N*-methyl-d-aspartate (NMDA) mediated toxicity (Wang et al., 2009), and promotes neuronal differentiation and survival (Islam et al., 2009). Evidence suggests that expression of IL-6 is beneficial following neuronal injury (Penkowa et al., 2000, 2003). While IL-6 is often undetectable in normal brain, its acute release in response to injury is well documented (Woodroofe et al., 1991; Taupin et al., 1993; Shohami et al., 1994; Williams et al., 2007; Ziebell et al., 2011). In rodent models, experimental TBI induces an increase in IL-6 mRNA expression in brain tissue after 1 h (Williams et al., 2007), and peaks in protein expression have been reported between 2 and 8 h after injury (Taupin et al., 1993; Shohami et al., 1994; Hang et al., 2004; Ziebell et al., 2011). In CSF, increases in IL-6 protein can be detected within 1 h, with peak expression between 2 and 5 h after an experimental brain injury (Woodroofe et al., 1991; Hans et al., 1999; Stover et al., 2000). The rapid increase in IL-6 expression following injury, and its maximal levels detected within a few hours makes this cytokine a promising candidate biomarker. However, it may have limited utility in stratification of patients since a similar temporal profile of IL-6 production has been demonstrated in most studies irrespective of the injury model used (weight-drop, FPI, CCI, or stab wound). The concentration of IL-6 in serum is rarely reported, but lower magnitude increases can be detected following injury (Maegele et al., 2007; Weckbach et al., 2012). Since the primary source of IL-6 following TBI originates in the brain, a limited ability of IL-6 to cross the BBB could explain in part this discrepancy. Indeed, studies in rodents and ovines have indicated that IL-6 has a limited ability to cross the BBB following a peripheral injection (Banks et al., 1994), and that a specific and saturable transport mechanism is involved in movement of IL-6 across the BBB (Threlkeld et al., 2010). These findings suggest that measurement of IL-6 in serum is unlikely to be truly indicative of brain concentrations, but rather the integrity of the BBB. Furthermore, since peripheral injuries can lead to changes in circulating IL-6 levels, models of “poly-trauma” are being developed (Maegele et al., 2005; Weckbach et al., 2012). Using these models, the specificity of serum IL-6 as a biomarker for brain injury has been found to be poor. While serum IL-6 concentrations were significantly higher in poly-trauma versus LFP or tibia fracture alone at 6 and 24 h after injury, there was no difference between the latter two groups (Maegele et al., 2007). In another model involving combined blunt bilateral chest trauma, lower limb fracture, and closed head injury (CHI), serum IL-6 was measured at 2 and 4 h after injury and found to be significantly higher in three-hit poly-trauma (CHI, chest trauma, and limb fracture) versus other groups at 4 h (Weckbach et al., 2012). Again there were no differences between single-hit groups or even combined CHI and chest trauma versus chest trauma and limb fracture. Therefore, current data on IL-6 in animal models is controversial. The rapid increase in IL-6 expression observed after brain injury and its peak within hours make it suited as a biomarker. Unfortunately, its limited ability to cross the BBB and apparent lack of ability to discriminate injury types may limit its usefulness.

### Clinical evidence

Under physiological conditions in humans, IL-6 expression in plasma is accepted as being 0–42 pg/mL, whereas in CSF there are only a few studies which have detected IL-6 under physiologically normal conditions (Froon et al., 1996; Kossmann et al., 1996; Maier et al., 2005). The largest of these studies measured IL-6 in the CSF of 113 patients, and reported IL-6 concentrations of 1–23 pg/mL (Maier et al., 2005). Following injury, IL-6 concentrations in CSF can reach concentrations as high as 35,500 pg/mL, but there appears to be a lot of variability in this response (Kossmann et al., 1996; Hillman et al., 2007). In addition, increases in serum IL-6 have been also been reported after TBI, with lower peak concentrations of 93–269 pg/mL (Winter et al., 2004; Chiaretti et al., 2005). Plasma levels of IL-6 greater than 100 pg/mL in the first 24 h following injury have been found to be associated with severe brain injury (Woiciechowsky et al., 2002). Data on the predictive ability of IL-6 in serum is limited and conflicting; in pediatric TBI serum IL-6 was reported to have no associations with neurological outcome (Kalabalikis et al., 1999), while others demonstrated that high IL-6 correlated with poor outcome (Arand et al., 2001; Woiciechowsky et al., 2002). More recently, measurements of serum IL-6 within 17 h of injury have been shown to identify patients at risk of developing elevated ICP (Hergenroeder et al., 2010). However, the authors also noted that lack of prognostic value of IL-6 for elevated ICP when patients also presented with extracranial injuries. Indeed, the presence of multiple injuries is common in TBI patients (Gennarelli et al., 1994; Meixensberger and Roosen, 1998) and should be considered when searching for or with the purpose of developing a biomarker. This is substantiated by evidence showing increased serum concentrations of IL-6 following orthopedic injury (Hergenroeder et al., 2010), burns (Agay et al., 2008), and exercise (Nybo et al., 2002; Febbraio and Pedersen, 2005). The poor predictive ability of IL-6 in the presence of multiple injuries is not surprising, and is corroborated by studies on experimental poly-trauma models described above. However, since the primary source of IL-6 in TBI are the cells of the CNS, including microglia, astrocytes, and neurons (Marz et al., 1998; Van Wagoner et al., 1999; Lau and Yu, 2001), more specific information on injury to the brain might be obtained from measurement of parenchymal cytokine production. This can be achieved using the technique of cerebral microdialysis, which can be adapted to recover cytokines and allow for continual sampling of brain parenchyma (Winter et al., 2002; Helmy et al., 2007). A wide range of cytokine concentrations can be measured in brain injured patients using this technique (Helmy et al., 2011). In a study involving 14 severe TBI patients, higher peak parenchymal levels of IL-6 were found to correlate with better GOS (Winter et al., 2004). Concentrations of NGF were also measured in this study, and while overall levels of NGF or IL-6 alone could not predict outcome, the ratio of NGF:IL-6 was significantly lower in survivors, and was correlated with GCS and GOS. In a more recent study including 16 patients with diffuse TBI, there was no relationship between parenchymal levels of IL-6 and ICP, brain tissue oxygenation, or the presence of brain swelling (Perez-Barcena et al., 2011). However, it must be noted that this study used averages of samples collected over 8-h periods, and could have missed some of he large-scale changes in cytokine concentrations known to occur in TBI. Furthermore, both of these studies suffered from a lack of statistical power, having relatively few patients. While further development of microdialysis techniques will undoubtedly provide us with some very useful information regarding the brain’s response to injury, it is limited by its invasive nature, the expense of the probes, and the highly region-specific information obtained.

In summary, IL-6 is highly sensitive to brain injury and can be easily detected in serum, although current data on its ability to predict outcome and its correlation with ICP are limited and inconclusive. Its inability to discriminate between brain damage and peripheral injuries may limit its usefulness in poly-trauma patients, but further development of parenchymal microdialysis and its use in combination with other biomarkers may prove fruitful.

## Interleukin-8/CXCL8 and Monocyte Chemoattractant Protein/CCL2

Interleukin-8 is a member of the CXC chemokine family (CXCL8), and is secreted by glial cells, macrophages, and endothelial cells (Aloisi et al., 1992; Nitta et al., 1992; Zhang et al., 2011). It is an important mediator for the activation and chemotaxis of neutrophils (Bickel, 1993). Early studies showed that IL-8 is released from astrocytes in response to other cytokines including IL-1β and TNF (Kasahara et al., 1991), both of which are expressed soon after brain injury (McClain et al., 1987; Woodroofe et al., 1991; Taupin et al., 1993). Increased IL-8 expression has been reported in many cancers (Xie, 2001), bacterial infections (Hirao et al., 2000), and is linked to cardiovascular disease (Apostolakis et al., 2009).

The monocyte chemoattractant protein-1 (MCP-1) or CCL2 is produced by astrocytes within hours after injury and its levels correlate with the amounts of recruited macrophages (Semple et al., 2010c). Since MCP-1 is regulated in an autocrine fashion, subsequent release of MCP-1 by infiltrated macrophages and microglia perpetuates cell migration in the injured brain. MCP-1 overexpression increased macrophage infiltration and neurological deficit in ischemia whereas its deletion attenuated infiltrates in brain injury, stroke, and multiple sclerosis models (Lu et al., 1998; Huang et al., 2001; Hughes et al., 2002; Chen et al., 2003).

### Laboratory evidence

Rodents lack a direct homolog for IL-8, but chemokine (CXC motif) ligand-1 (CXCL-1), CXCL-2, CXCL-5, and CXCL-6 appear to be functional homologs, residing in the same gene cluster as IL-8 (human chromosome 4q13.3) and contributing to neutrophil recruitment in a number of animal models. In rat CCI, the expression of the chemokine CXCL-1 peaks at 4 h, and is reduced by 12 h, but remains elevated versus control for up to 7 days after injury (Dalgard et al., 2012). In our model of CHI, the synthesis of MIP-2 as well as other chemokines (MCP-1, MIP-1α, RANTES, and KC) in the cortex increased at 4–12 h preceding the infiltration of neutrophils and macrophages which peak at 24–48 h and 4–7 days, respectively. MIP-1α and MIP-2 concentration was reduced in the brain of TNF-KO mice after TBI, implying a role for TNF in regulating their expression (Otto et al., 2002). Amplified expression of chemokine receptors CXCR2 and CCR2 was localized on infiltrating neutrophils and macrophages, respectively at 1 and 4 days post-TBI (Otto et al., 2001; Semple et al., 2010b). While neutrophils depart by 1 week from the injured brain, the accumulation of macrophages persists over 4 weeks (Semple et al., 2010a). The prolonged presence of activated leukocytes within the pericontusional tissue is likely detrimental due to their ability to secrete neurotoxins leading to delayed neuronal death. The role of MCP-1 and IL-8/MIP-2 in secondary brain degeneration and neurological function was recently explored using MCP-1-KO and CXCR2-KO mice. The most striking data in MCP-1-KO mice showed a significant reduction in lesion volume, neuronal loss and macrophage accumulation up to 46% over 4 weeks after TBI as opposed to wild-type mice (Semple et al., 2010a). Improved brain damage resulted in faster neurological recovery from 1 to 4 weeks. In CXCR2-KO mice, an 80% decline of neutrophil infiltration occurred at 12 h after TBI and coincided with reduced lesion and neuronal loss over wild-type controls (Semple et al., 2010b).

### Clinical evidence

In humans, IL-8 is detected at very low levels in the CSF and plasma of healthy individuals. Physiological plasma concentrations are 5–18 pg/mL, and in CSF are 5–72 pg/mL (Maier et al., 2005). Following TBI there is an increase in IL-8 concentration in serum and CSF (Kossmann et al., 1997; Morganti-Kossman et al., 1997). IL-8 appears to peak early following a TBI, with mean levels up to 29,000 pg/mL reported in CSF (Whalen et al., 2000; Kushi et al., 2003a). Increases in plasma levels of IL-8 following brain trauma have been reported, but are of lower magnitude and more variable. Peak concentrations of approximately 100 pg/mL are commonly demonstrated in severe TBI (Kossmann et al., 1997; Mussack et al., 2002; Kushi et al., 2003a). Increased IL-8 in CSF has been associated with mortality. In one study, CSF was obtained from 27 children who had sustained a severe TBI, 7 with meningitis, and 24 children with neither diagnosis. The increase in IL-8 levels in children with TBI was of similar magnitude to children with meningitis. Heightened IL-8 expression persisted for several days, and a significant correlation was found between IL-8 and mortality (Whalen et al., 2000). In another study, Gopcevic et al. collected blood and CSF samples at time of admittance from 20 adults who had sustained severe TBI, 10 of which died. They showed that plasma IL-8 was significantly lower in survivors versus non-survivors (71 and 111 pg/mL respectively), however, CSF IL-8 concentrations were not different. They also showed that IL-8 had a prognostic value for GCS, patient age, and Acute Physiologic and Chronic Health Evaluation (APACHE II; Gopcevic et al., 2007). Although plasma levels of IL-8 are considerably lower than CSF, several studies have found correlations between peripheral IL-8 and outcomes. Mussack et al. (2002) measured serum IL-8 at intake and 12 h later in 20 TBI patients and found a significant correlation of increased IL-8 levels 12 h after admission with outcomes assessed by GOS. In another study, serum concentrations of IL-8 72 h after TBI were significantly higher in non-survivors versus survivors (Kushi et al., 2003a). Most recently, Lo et al. took blood samples from 28 pediatric TBI patients at precisely 24 h following injury and correlated serum biomarker levels with unfavorable outcomes 6 months later (GOS < 4). Increased serum IL-8 was found to correlate with unfavorable outcome. Furthermore, when combined with GCS and increased specificity, an sensitivity was observed (Lo et al., 2010). The weakness of this study was that only 4 patients had an unfavorable outcome, nonetheless, it demonstrates the potential for using paired markers to predict outcomes with greater accuracy.

Our group has demonstrated that in CSF samples from 21 severe TBI patients, MCP-1 concentrations increased to 19,000 pg/mL on day 1, falling to approximately 3000 pg/mL from day 3 onward (Semple et al., 2010a). The rapid peak in CCL2 levels in CSF and its elevated expression in the CSF for several days merit investigation of this cytokine as a potential biomarker for TBI.

Upregulation of both IL-8 and MCP-1 at mRNA and protein level in post-mortem human brain, underlining the relevance of the chemokine network in human TBI (Frugier et al., 2010). Specifically, a 140-fold increase in IL-8 mRNA was detected in the injured brain compared to control, being the mediator with the strongest increase, while MCP-1 mRNA was increased by almost 20-fold (unpublished data). Overexpression of chemokines in these human samples was associated with tissue infiltration of CD68-positive macrophages and GFAP-positive reactive astrocytes (Frugier et al., 2010). Combined, these experimental studies provide compelling evidence that signaling through chemokine networks contributes to secondary tissue and neurological damage and could be considered in future studies assessing their meaning as biomarkers of TBI.

## Other Cytokines

The cytokines described above are the best characterized in terms of their role in neuronal injury and their potential as markers for TBI, however, there are a large number of less-well characterized immune modulators that could be useful TBI biomarkers.

Another potential biomarker is GM-CSF, a pro-inflammatory cytokine that is expressed in the CNS by neurons, astrocytes, and microglia (Franzen et al., 2004). GM-CSF is secreted by vascular endothelial cells, it crosses the BBB and can be detected in CSF (Coxon et al., 1999). We have found this cytokine to be significantly upregulated in post-mortem tissue from TBI patients that died 6–122 h after injury (Frugier et al., 2010). More recently we have found that GM-CSF is more highly expressed in the CSF of patients suffering from secondary hypoxia versus normoxic TBI patients, and is also increased in diffuse TBI versus focal TBI (unpublished results). The different levels of expression of this cytokine between injury types merits further investigation of this molecule as a TBI biomarker.

Also of potential interest is endothelial monocyte-activating polypeptide II precursor (p43/pro-EMAP II), a pro-inflammatory cytokine linked with apoptosis (Knies et al., 1998; van Horssen et al., 2006). In a high-throughput immunoblotting screen of 998 proteins in rats 24 h after ischemic injury versus penetrating TBI differential expression was found in only the cytokine p43/pro-EMAP II (Yao et al., 2008). In a subsequent study, tissue, blood, and CSF concentrations of this cytokine were shown *in vivo* to discriminate between ischemic brain injury and TBI modalities (Yao et al., 2009). Specifically, Yao et al. found that p43/pro-EMAP II is constitutively expressed in the brain of naïve rats, but significantly increases in CSF and plasma 24-h after penetrating ballistic-like brain injury, whereas a significant decrease was found in CSF and plasma following middle cerebral artery occlusion. Western blotting of brain tissue at 6, 24, 48, and 72 h showed similar increases in p43/pro-EMAP II expression at all time-points post-TBI, and significant decreases in expression following ischemic brain injury. Immunohistochemistry revealed that changes in p43/pro-EMAP II levels were due to changes in neuronal expression and decreases did not represent neuronal loss. Taken together, these data show that the little-studied p43/pro-EMAP II cytokine has potential to be a useful brain-specific biomarker.

## Other Markers of Inflammation

In addition to cytokines, several other molecules could potentially be useful as biomarkers of brain injury. For example, activation of the JAK/STAT pathway by IL-6 regulates GFAP expression. It is well established that GFAP expression increases in serum following TBI (Missler et al., 1999; van Geel et al., 2002). In severe TBI patients, serum GFAP levels have been shown to be able to predict mortality and outcome (Pelinka et al., 2004a,b; Nylen et al., 2006; Vos et al., 2010). More recently, GFAP-BDPs in serum of mild and moderate TBI patients within 4-h of injury has been found to correlate with injury severity (GCS), and maybe be associated with CT lesions (Papa et al., 2012). In a recent study it was found that combining measurements of GFAP in CSF and serum with the IMPACT Outcome Calculator a significant improvement in outcome prediction could be achieved (Czeiter et al., 2012). In addition, the expression of αII-spectrin breakdown product 145 (SBDP145) was measured in CSF and correlated significantly with 6-month mortality (Czeiter et al., 2012). Another study measured SBDPs in the CSF of severe TBI patients at the time of admission and every 6 h thereafter for up to 7 days (Mondello et al., 2010). It was shown that in addition to an increased expression of SBDPs in all TBI patients versus controls, there was a significant correlation of SBDP145 with injury severity (assessed by GCS). Furthermore, levels of SBDP145 and SBDP120 were significantly higher in patients that died, suggesting that these markers may be able to predict mortality (Mondello et al., 2010). Another marker of note is the microtubule-associated proteins (MAP-2), which are neuronal specific proteins found in dendrites (Drewes et al., 1998). Laboratory studies in models of ischemic and traumatic injury have established that MAP-2 expression is lost from injured brain regions, and increases in MAP-2 expression can be detected in serum shortly after injury (Kitagawa et al., 1989; Posmantur et al., 1996; Park et al., 2012). More recently, it has been shown that serum MAP-2 expression measured 6 months after severe TBI is still elevated. Furthermore, increased serum MAP-2 expression correlates with better outcome (GOSE), and was found to be higher in non-vegetative state patients versus vegetative state patients (Mondello et al., 2012). This suggests that MAP-2 has potential as a marker for outcome prediction, and increased serum MAP-2 expression may signal the emergence of higher cognitive function in severe TBI patients. Lastly, the inflammasome is responsible for the production of mature IL-1β and IL-18, and may therefore provide us with useful brain injury biomarkers. In a recent study, CSF was collected from 23 patients who had suffered a moderate or severe TBI, and levels of inflammasome proteins were measured (Adamczak et al., 2012). Apoptosis-associated speck-like protein containing a caspase recruitment domain (ASC), caspase-1, and Nacht leucine-rich-repeat protein-1 (NALP-1) were all elevated in the CSF of TBI patients versus controls. Furthermore, all 3 proteins correlated significantly with outcome (GOS at 5-months; Adamczak et al., 2012).

## Comparable Studies in Spinal Cord Injury

Development of biomarkers for CNS injury has focused almost exclusively on brain injury, however, markers for spinal cord injury (SCI) are also much needed. MRI after SCI can be used to detect hemorrhage, transaction, and lesions, but is not always the best method for predicting outcomes. Neurological assessment has been shown to be predictive for outcome, but cannot be administered within the first critical hours of injury (Lammertse et al., 2007). Biomarkers for SCI could allow clinicians to make earlier prognoses and decide on the best course of treatment. Research into biomarkers of SCI is very limited and has focused almost exclusively on S100β and NSE. Laboratory research has shown that in the CSF, expression of S100β, NSE, and neurofilament protein (NFL) are increased (Skouen et al., 1999; Nagy et al., 2002; Cao et al., 2008). A similar increase can be measured in the serum of animals subjected to an experimental SCI (Ma et al., 2001; Loy et al., 2005; Cao et al., 2008). There is some evidence from these studies that NSE and S100β can distinguish between injury severities. Both NSE and S100β expression was shown to correlate with injury severity in a rat weight-drop model of SCI (Cao et al., 2008). However, a similar study failed to find a difference in serum or CSF NSE expression between graded injury levels (Loy et al., 2005). Human studies into SCI biomarkers have focused on detecting ischemic injury in patients undergoing thoracoabdominal aortic aneurism surgery (van Dongen et al., 1998, 1999; Kunihara et al., 2001; Winnerkvist et al., 2007), and other studies have looked at S100β or NSE after surgery for lumbar disk herniation (Brisby et al., 1999), spinal epidural empyema (Marquardt et al., 2004b), or paresis due to spinal metastasis (Marquardt et al., 2004a). Data from these studies is inconclusive with regards to either S100β or NSE being useful as markers of ischemic injury. While several studies have reported increases in expression of these biomarkers, little is known with regard to how this relates to outcome. In the case of spinal epidural empyema or paresis due to spinal metastasis, longer periods of elevated serum S100β were related to unfavorable outcome (Marquardt et al., 2004a,b). Lastly, two studies have assessed biomarkers for human traumatic SCI. The first measured GFAP and NFL expression in CSF of patients with traumatic SCI, and although both markers were increased, no statistical analysis was done and only six patients were included (Guez et al., 2003). The second study took CSF from 27 patients with complete or incomplete SCI and measured protein expression of IL-6, IL-8, MCP-1, S100B, and GFAP (Kwon et al., 2010). All of the markers were increased in SCI patients, and there were significant differences in expression of each marker between injury severities. Furthermore, a model was developed that accurately predicted injury severity and outcome at 6-months post-injury. The results of this study suggest that biomarkers may be useful in SCI patients.

## Discussion

Brain injury biomarkers promise to improve patient diagnosis, management and outcomes, and aid in the development of novel therapeutics. The now well accepted inflammatory response that occurs in the injured brain has the potential to offer clinicians a number of markers that could provide specific information on the injury. Experiments in animal models of TBI have revealed a plethora of inflammatory mediators that are expressed in the brain following injury. Many of these exhibit rapid changes in expression, reaching peaks of over 1000 orders of magnitude greater than physiological levels within hours of injury. The magnitude, timing, and duration of expression of these mediators might be able to provide not only information about the injury but also of the complexity deriving from the combination of multiple insults. For example, in a rat Marmarou model of diffuse brain injury, TNF production is of a higher magnitude, and IL-1β expression is heightened and prolonged, when the injury is followed by a 30-min period of hypoxia (Yan et al., 2011). This suggests that cytokines may indeed reveal various degrees of brain damage with overlapping insults.

Translating information from animal studies into clinically relevant concepts can prove challenging, since human TBI is a very varied condition that is often accompanied by additional peripheral injuries, especially if we consider that animal models mostly reproduce a single form of TBI. Additionally, the human brain is gyroencephalic, and with a larger mass than the rodent brain it also presents a different ratio of white to gray matter. The development of inflammatory mediators as reliable markers of brain injury is jeopardized by the fact that peripheral injuries induce an immune response that may mask or be indistinguishable from the inflammatory response occurring in the brain. Indeed, several studies have noted that in the presence of multiple injuries, markers of inflammation cannot discriminate for the presence of brain injury (Hensler et al., 2000; Dziurdzik et al., 2004; Shiozaki et al., 2005; Hergenroeder et al., 2010). To this end, models of so called “poly-trauma” are being developed, which combine experimental TBI with peripheral injuries (Maegele et al., 2005; Weckbach et al., 2012). These can then be used to search for specific brain injury markers. Another consideration is that in most animal studies cytokine concentrations are measured directly in homogenized brain tissue rather than in CSF or blood samples. While this gives very useful description of the specific response of the brain to injury, such measurements are not possible in the clinic and if done they would have little relevance when considering the definition of a biomarker for early diagnostic and prognostic significance. An additional consideration should be differences in immune activation occurring in the species utilized in modeling TBI. Higher brain TNF levels have been reported in rats as compared to mice in equivalent severity of cerebral ischemia (Schroeter et al., 2003), so different responses between rodents and humans must be also expected. Even within the human population there is evidence that polymorphisms in cytokine genes could affect not only outcome, but also the subject level of cytokine synthesis in response to injury (Hadjigeorgiou et al., 2005; Uzan et al., 2005). Finally, the redundancy inherent in the inflammatory response is important to consider, since it makes it quite likely that in a genetically diverse population of humans a degree of variability will be observed in cytokine responses.

In studies that have concomitantly measured cytokine levels in CSF and serum differences in concentration and even timing of peaks has been observed (Kossmann et al., 1995; Csuka et al., 1999; Shiozaki et al., 2005). This undoubtedly reflects the compartmentalization of the CNS from the periphery and the limited diffusion of cytokines out of the brain parenchyma and vice versa. Changes in BBB compliance following TBI are also known to occur, and may affect the CSF:serum ratio of some cytokines (Kossmann et al., 1995; Csuka et al., 1999), as well as sequestration of cytokines by the liver (Wu and Pardridge, 1999). Nonetheless, many groups have been able to successfully detect significant increases in inflammatory markers in the blood following TBI and established associations or correlations with other injury parameters and outcomes. Among the cytokines, perhaps the most promising to date is IL-6, since 100-fold increases can be readily measured in serum following TBI (Winter et al., 2004; Chiaretti et al., 2005). IL-6 levels have been correlated with ICP (Hergenroeder et al., 2010), outcome (Arand et al., 2001; Woiciechowsky et al., 2002), but not in multi-trauma patients. To overcome this problem sampling for cytokine biomarkers could be done in CSF or in the brain parenchyma itself by microdialysis to get a CNS specific measurement of the immune response. Since ICP monitoring and management is common in TBI patients, CSF samples could be obtained from severely injured patients. Changes in CSF cytokine concentrations can be orders of magnitude greater than serum concentrations, and do not appear to be affected by peripheral injuries. However, data is still mixed with regard to the ability of cytokines to distinguish injury severity and type, predict or correlate with ICP, or to predict outcome characteristics. Further study of the cellular origin and biochemical meaning of raised or lowered level of a specific cytokine may help to interpret these data.

Given the problems discussed above, improved sensitivity may be achieved by combining a multitude of biomarkers with conventional neuroimaging techniques, and neurological scoring (e.g., GCS, GOS/E). Novel cytokine biomarkers could be measured in parallel with “classical” TBI biomarkers such as S100β and NSE to improve sensitivity. We have shown that CSF concentrations of sICAM-1 correlate well with tissue and BBB damage, giving an indication of the degree of immunologic activation in the injured CNS (Pleines et al., 1998), and in a subsequent publication measured sICAM-1 together with well known TBI biomarkers (Pleines et al., 2001). In the latter publication we showed that mean CSF protein concentrations of S100β correlate with IL-6, contusion size assessed by CT, and GOS, while serum S100β correlates with contusion size and GOS. In this study we also showed that NSE serum levels correlate with IL-6, and that NSE levels in CSF correlate with sICAM-1 and contusion size (but not GOS). Taken together, the correlation of serum S100β with contusions size and outcome shows that it reflects the extent of injury well, but NSE and cytokine biomarkers give a better indication of the degree of inflammation in the brain. Other groups have gone one to show that by combining pairs of biomarkers including IL-6, IL-8, S100B, and NSE, a higher degree of outcome predictability can be achieved versus any single biomarker (Winter et al., 2004; Berger et al., 2009; Lo et al., 2009). Similarly, combining GCS score with a single biomarker such as IL-8 also improves outcome predictability (Lo et al., 2010). Recently developed methods for predicting outcomes based on age, motor score, pupillary reactivity, and CT characteristics (IMPACT, Steyerberg et al., 2008) have been shown to benefit from the inclusion of brain injury biomarkers (Czeiter et al., 2012). Although inflammatory markers were not included in that study, several cytokines have been shown to have power to predict outcome, including IL-1β, IL-6, IL-8, and IL-10 (Bell et al., 1997; Whalen et al., 2000; Arand et al., 2001; Mussack et al., 2002; Woiciechowsky et al., 2002; Kushi et al., 2003b; Chiaretti et al., 2005; Shiozaki et al., 2005; Gopcevic et al., 2007; Kirchhoff et al., 2008).

In addition to being useful in prediction of changes in ICP, mortality, or 6 month outcomes, biomarkers could be used to categorize patients based on specific pathophysiological processes occurring in the injured brain. This information could help to provide individualized treatment based on the specific type or severity of injury. Using a rat model of diffuse injury we have shown that an additional hypoxic insult enhances cortical production of the cytokines IL-1β, IL-6, and TNF (Yan et al., 2011). In human studies, correlations have been found between injury severity and concentrations of a specific cytokine, such as IL-1β (Aly et al., 2006), IL-6 (Kossmann et al., 1996; Kalabalikis et al., 1999; Woiciechowsky et al., 2002; Chiaretti et al., 2005), and IL-10 (Neidhardt et al., 1997). An improved understanding of the roles of these cytokines in the secondary injury process may pave the way for targeted treatment strategies tailored specifically to the patient. Measurement of these cytokines could also be used to track the effects of a potential pharmacological treatment. For example, methylprednisolone (MP) has anti-oxidant and anti-inflammatory effects and is used in the treatment of SCI. Administration of MP to rats subjected to experimental SCI or subjected to an inflammatory stimulus has been found to reduce TNF expression (Buttini et al., 1997; Xu et al., 1998), and expression of other cytokines (Fu and Saporta, 2005). Another potential treatment is the tetracycline antibiotic, minocycline, which has been found to have anti-inflammatory effects. We have shown that IL-1β expression is significantly reduced in mouse brain subjected to CHI when minocycline is administered (Bye et al., 2007). Although interestingly, there was no significant reduction in other cytokines, including TNF, IL-6, G-CSF, MCP-1, and MIP-2.

The future for inflammatory mediators as biomarkers for use in TBI is still uncertain, in large part due to their lack of specificity. However, development of animal models of multi-trauma, the use of two or more markers, and new sampling techniques may overcome this problem. There is also a need to gain a more complete understanding of the temporal expression profile of each cytokine in specific types and severities of injury, since data is currently limited. The use of multiplex assays now allows for simultaneous measurement of several cytokines in brain samples and is providing useful information in this regard (Yan et al., 2011; Dalgard et al., 2012). In addition, the use of microdialysis technology in patients, while invasive and expensive, has potential to provide us with continual cytokine concentrations within the brain parenchyma itself (Winter et al., 2002; Helmy et al., 2007). Intraparenchymal measurement of this kind negates the need to consider BBB disturbances and may be more biologically relevant.

In conclusion, the monitoring of the inflammatory process has the potential to provide specific information on the injury and make predictions about probable outcomes. Several cytokines have shown potential in this area, but a more complete understanding of their specific roles and expression profiles is needed.

## Conflict of Interest Statement

The authors declare that the research was conducted in the absence of any commercial or financial relationships that could be construed as a potential conflict of interest.
